# Inducible Knockout of the Cyclin-Dependent Kinase 5 Activator p35 Alters Hippocampal Spatial Coding and Neuronal Excitability

**DOI:** 10.3389/fncel.2018.00138

**Published:** 2018-05-17

**Authors:** Eriko Kamiki, Roman Boehringer, Denis Polygalov, Toshio Ohshima, Thomas J. McHugh

**Affiliations:** ^1^Laboratory for Molecular Brain Science, Department of Life Science and Medical Bioscience, Waseda University, Tokyo, Japan; ^2^Laboratory for Circuit and Behavioral Physiology, RIKEN Center for Brain Science, Wako-shi, Japan

**Keywords:** cyclin-dependent kinase 5, p35, hippocampus, CA1, spatial coding, neuronal excitability, *in vivo* electrophysiology, place cells

## Abstract

p35 is an activating co-factor of Cyclin-dependent kinase 5 (Cdk5), a protein whose dysfunction has been implicated in a wide-range of neurological disorders including cognitive impairment and disease. Inducible deletion of the p35 gene in adult mice results in profound deficits in hippocampal-dependent spatial learning and synaptic physiology, however the impact of the loss of p35 function on hippocampal *in vivo* physiology and spatial coding remains unknown. Here, we recorded CA1 pyramidal cell activity in freely behaving p35 cKO and control mice and found that place cells in the mutant mice have elevated firing rates and impaired spatial coding, accompanied by changes in the temporal organization of spiking both during exploration and rest. These data shed light on the role of p35 in maintaining cellular and network excitability and provide a physiological correlate of the spatial learning deficits in these mice.

## Introduction

Cyclin-dependent kinase 5 (Cdk5) is a proline-directed serine/threonine kinase structurally homologous to the Cdk family members which modulate the cell cycle. It is unique, however, in that it is active in post-mitotic neurons and is regulated by two brain specific co-factors, p35 and p39 (Tsai et al., 1994; Tang and Wang, 1996; Takahashi et al., 2005). Cdk5 plays numerous critical roles in the developing mammalian nervous system, influencing the migration and differentiation of neurons and oligodendrocytes, intracellular trafficking and axonal elongation (Ohshima et al., 1996, 2001; Tanaka et al., 2001; Smith et al., 2003; Miyamoto et al., 2007). In the adult brain, the Cdk5/p35 complex has been shown to function both pre- and post-synaptically as a regulator of synaptic transmission and plasticity. Pre-synaptically, it can impact both endo- and exocytosis and may work as a negative regulator of transmitter release (Cheng and Ip, 2003; Cheung et al., 2006). Post-synaptically, it influences dendritic spine density (Mita et al., 2016) and targets many proteins in the post-synaptic density, including N-methyl-D-aspartate receptors (NMDARs), which are required for synaptic plasticity (Hawasli et al., 2007; Plattner et al., 2014). Given this myriad of diverse functions, it is perhaps not surprising that dysfunction in the Cdk5 pathway has been linked to cognitive impairments and a number of pathologies, including epilepsy (Wenzel et al., 2001; Putkonen et al., 2011) and Alzheimer’s disease (AD; Lew et al., 1994; Patrick et al., 1999; Yoo and Lubec, 2001).

Activation of Cdk5 in neurons is tightly regulated through its interaction with the co-factors p35 and p39 (Tsai et al., 1994; Tang and Wang, 1996; Takahashi et al., 2005). Previous work has demonstrated that the loss of p35 in the embryo leads to severe developmental abnormalities, resulting in an inversion of the cortical layers in the adult brain (Chae et al., 1997). While these animals were found to have deficiencies in hippocampal-dependent spatial learning and alterations in *in vitro* synaptic plasticity, interpretation of these phenotypes is complicated as a result of the changes in the gross anatomy. More recently, using an inducible knockout approach (p35 cKO mice), it was found that loss of p35 in the adult mouse results in learning and memory deficits, as well as morphological and physiological changes, including alterations in synaptic plasticity (He et al., 2014; Mishiba et al., 2014). These conditional knockout mice demonstrate severe impairments in spatial learning in the water maze, as well as changes in physiology, anatomy and biochemistry within the hippocampus (Mishiba et al., 2014; Mita et al., 2016). CA1 pyramidal cells in p35 cKO mice have significantly fewer dendritic spines and are less sensitive to Schaffer collateral input arriving from CA3; changes that both suggest possible decreases in neuronal excitability. However, CA3-CA1 synapses also show impaired long-term depression (LTD), a phenotype that may prevent activity dependent regulation of synaptic strength. Despite the wealth of *in vitro* data on CA1 pyramidal cell morphology and synaptic physiology in mice lacking p35, the impact of these changes on *in vivo* physiology remains unknown.

The hippocampus is one of the most well characterized and intensely studied regions of the mammalian brain. A rich body of anatomical and behavioral literature suggests it plays a key role in the acquisition, storage and recall of certain types of memory, including the memory for space and context (Jarrard, 1993). These data, coupled with the observation of O’Keefe and Dostrovsky (1971) that single hippocampal neurons increased their firing rate whenever a rat traversed a particular region of a chamber (the cell’s “place field”), triggered the proposal that these cells allow the hippocampus to serve as the neural substrate of a “cognitive map” and earned them their moniker, “place cells” (O’Keefe and Nadel, 1978). In addition to this firing rate based spatial code, the spiking of hippocampal pyramidal cells is temporally modulated by oscillations in local field potential (LFP), which impact communication and information transfer both within and outside the circuit (Ahmed and Mehta, 2009; Middleton and McHugh, 2016). The hippocampal LFP has two dominant states, the theta rhythm (6–12 Hz), which temporally organizes spiking during memory encoding (O’Keefe and Recce, 1993; Buzsáki, 2002) and sharp-wave ripples (SWRs; Buzsáki, 2015), short ~140 Hz oscillations seen during consummatory behaviors and slow-wave sleep, critical for memory consolidation (Girardeau et al., 2009; Nakashiba et al., 2009; Ego-Stengel and Wilson, 2010). In the last two decades, *in vivo* recordings in freely behaving genetically-modified mice have been key in understanding how alteration in synaptic plasticity and intracellular signaling impact these codes for space (McHugh et al., 1996; Cho et al., 1998; Cacucci et al., 2007; Resnik et al., 2012; Cheng and Ji, 2013). Here, we recorded the spiking of CA1 pyramidal cells and the concurrent LFP in the dorsal hippocampus of freely behaving p35 cKO and control mice to better understand the impact of the loss of p35 on network function.

## Materials and Methods

### Subjects

#### Mice

The experimental protocols were approved by RIKEN Animal Care and Use Committee and the Institutional Animal Care and Use Committees of Waseda University. All efforts were made to minimize the number of animals and their suffering that accompanies the experiments. Mice were housed in transparent Plexiglas cages which were stored in ventilated racks and were maintained in a humidity and temperature-controlled room. The 12 h light/dark cycle (lights on from 8:00 to 20:00) was maintained in a standard manner. Prior to stereotaxic surgery, animals were housed in groups of up to five and food and water were provided *ad libitum*. Animals were singly housed after intracranial implantation of electrodes. All animals used in this experiment were male, p35 cKO mice (homozygous for the p35-flox allele and carrying the CACGCre-ER transgene (Jackson stock #004682)) and *fl*p35 littermate controls (homozygous for the p35-flox allele) between 2 months and 4 months of age. This mouse line was generated and maintained on a C57BL/6 background and genotyped as previously reported (He et al., 2014; Mishiba et al., 2014). All experiments were conducted during the animal’s light cycle.

#### Preparation and Administration of Tamoxifen

Dimethyl sulfoxide (Sigma Life Science) was dissolved in methanol (Nacalai Tesque) in a ratio of 5:2 for obtaining a MetDMSO mixture. Twenty milligrams of tamoxifen (Sigma Life Sciences) was then dissolved in a 10-fold dilution of MetDMSO with corn oil. To ensure that all the tamoxifen has dissolved, the solution was vortexed vigorously. The tamoxifen solution was divided into 500 μL aliquots and stored at −20°C for stability and to prevent unnecessary light exposure. Mice were administered the drug at a dose of 3 mg/40 g of body weight via oral gavage (Fine Science Tools Muromachi) daily for 3 days. A new aliquot was used daily and any unused portion of the aliquot was disposed off. p35 cKO and control mice were given identical tamoxifen treatments.

### *In Vivo* Electrophysiology

#### Construction of Tetrode Microdrive Arrays

Custom microdrive arrays were constructed with support of Advanced Manufacturing Support Team, RIKEN Center for Advanced Photonics, Japan. Each drive consists of two parallel rows of four independently adjustable tetrodes (TTs), constructed from 14 μm Nichrome wire. All the TTs were gold plated to reduce their impedance to around 200–250 kΩ. The TTs were then implanted running along the CA3 to CA1 axis of the dorsal hippocampus.

#### Microdrive Array Implantation and Tetrode Adjustment

Avertin (2,2,2-tribromoethanol; Sigma-Aldrich, 476 mg/kg, i.p.) was used to anesthetize the mice prior to surgery. The mice were placed into a stereotactic frame (Kopf) and microdrive array was implanted over the craniotomy based on the coordinates of CA1 of dorsal hippocampus standardized at the bregma (Anterior-Posterior (AP): −1.6 mm, Medial-Lateral (ML): −1.0 mm, Dorsal-Ventral (DV): −1.3 mm). Two days post-surgery, individual TTs were lowered over several days until all of them reached *stratum pyramidale* (SP) layer of the hippocampus. During the adjustment, the mice were kept in a small (15 cm diameter) sleep/rest box. TTs were then adjusted in small steps until ripple events began to appear in the acquired LFP, indicating proximity of the SP for maximum cell yield. LFP and spike recording were then commenced.

#### Experimental Protocol

Mice were habituated to a linear track (LT: 140 cm × 10 cm, semi-transparent gray 15 cm walls) prior to microdrive implantation for 3 days. Once electrodes were in place, subjects were recorded on this familiar LT (20 min for minimum 10 Laps). Additional sleep/wakeful rest (60 min) session was recorded in the adjusting box immediately before and after the LT. The animals were recorded for three consecutive days. Mice were then exposed to one of novel LTs (LT2: 140 cm × 10 cm brown walls 15 cm in height, electric tape flooring, almond odor; LT3: 140 cm × 10 cm black striped walls 15 cm in height, coated plastic flooring with banana odor; LT4: 140 cm × 10 cm black walls with green anti-slip coated flooring, acetic acid odor) for 20–30 min. While only single novel track was used per every recording session, all the mice would have experienced all of them prior to fixation. The mouse was put in the “rest/sleep” box again (20 min) and then exposed back to the “familiar track” (LT1) for 20–30 min. The recording session was finished with subsequent 1-h rest/sleep trial.

After all the recordings were carried out, the mice were transcardially perfused with 4% paraformaldehyde (PFA) in 0.1 M sodium phosphate buffer (PBS). Terminal anesthesia was first performed by Avertin injection (1 mL) and TT positions were marked by with electrolytic lesions (30 μA current, 5 s for each TT). The brain was then post-fixed for another 24 h in 4% PFA. Fifty micrometers coronal sections were sliced with a vibratome (Leica). The slices were visually checked using a standard light microscope to confirm appropriate electrode implantation, both for location and depth.

#### Data Recording, Pre-processing and Unit Isolation

Data were acquired using a 32-channel DigitalLynx 4SX acquisition system and Cheetah v5.6.0 software (Neuralynx). Signals were sampled at 32,000 Hz and filtered between 0.6–6 kHz for spike detection. Mouse’s head position and direction were concurrently tracked using a pair of red/green light emitting diodes (LEDs) affixed to the microdrive array. Data files of each dataset (single recording session) were first split by trial timestamps (“sleep”/“track”) using EventSessionSplitter software (Neuralynx). Artifacts of animal’s head position values occurred due to obscuring of LEDs by tether cable were filtered out using custom written algorithm. Positional data were smoothed with a Gaussian kernel of 0.05 standard deviation (SD) width. Then each of the single units was manually isolated by drawing cluster boundaries around the 3D projection of the recorded spikes, presented in SpikeSort3D software (Neuralynx). In order to assess stability of spatial coding the same cells were tracked across all the trials (sleep/familiar LT/novel LT) of each recording session. In order to ensure that manually clustered spikes belong to a valid single units, several clustering quality criteria were applied, including: (a) clusters which have more than 0.5% of their spikes with an inter-spike interval (ISI) less than 2 ms; (b) fired less than 50 spikes per trial; and (c) isolation distance (ID) measure was less than 10 (Schmitzer-Torbert et al., 2005). Cells which met one or more of the above criteria were excluded from further analysis. In this study only those units which were classified as pyramidal cells were used. Normally, spikes generated by such cells have an average spike width >200 μS and a complex spike index measure ≥5 (CSI; McHugh et al., 1996). Animal’s velocity was computed on a per-frame basis by using the recorded position coordinates and corresponding timestamps and smoothed with a 2.5 SD Gaussian Kernel. Further analysis was done by using custom scripts written in MATLAB software (MathWorks, Natick, MA, USA).

#### Firing Rate Map Properties

Firing rate maps were generated based on a calculation of how many spikes fell into each of the 1 cm × 1 cm spatial bin divided by the total occupancy time of that bin. The maps were then smoothed with a 1 SD Gaussian Kernel, excluding non-visited bins. The maximum firing rate value for each of the rate map was defined as the peak firing rate, while the mean firing rate was computed by finding the average rate calculated by dividing the number of spikes by the period of duration at which the velocity was greater than 2 cm/s and taking the mean of these values. All analyses on place cells and place field on the familiar track were restricted to the first run session of the day to avoid duplication of the same neurons.

#### Place Field Detection and Quantification

A place field was determined as a set of contiguous spatial bins that surround the bin containing the maximum firing rate. For a cell to be considered as a place cell, they were required to have a minimum field size of six bins (each bin size = 1 cm × 1 cm), with a mean firing rate >0.2 Hz and a peak firing rate >1.0 Hz with positive signal to noise ratio. The size of the place field was measured in terms of number of spatial bins that contained place cell field firing that was higher than 20% of the peak firing rate. Percent of sampled space was defined as a percentage of spatial bins belonging to the main place field of any given pyramidal cell relative to the total number of spatial bins visited by the animal. Place field stability in the familiar track was accessed by calculating spatial cross-correlation (Pearson’s *R* across spatial bins) between place fields of the same single units recorded during first and second exposure of the mouse to the familiar track.

#### Spatial Information (Bits/Spike)

Spatial information represented in bits/spike indicates the measure of extent to which the firing of the cell can be used to predict the position of the animal. The spatial information that each of the cell carries can be calculated as previously reported (Skaggs et al., 1993). Briefly, the equation used was SI = Σ (P_spk_(i) * log 2 (P_spk_(i)/P_occ_(i))), where P_spk_(i) represents the probability of spiking in bin “i” and P_occ_(i) refers to the occupancy probability in bin “i.” P_spk_ and P_occ_ are computed from the rate and occupancy maps in the corresponding order.

#### Complex Spike Index

The CSI indicates the percentage of cells that qualify the properties of complex spike (McHugh et al., 1996). Complex spike burst is a characteristic *in vivo* pattern of action potential generation in the CA1 pyramidal cells. It defines the electrophysiological signature of hippocampal pyramidal cells which may represent an important form of information coding in the hippocampus. Quantitatively, it is defined as CSI = 100 * (pos − neg) where “pos” is the number of ISIs which contribute positively to CSI (preceding spikes have larger amplitudes followed by spikes with smaller amplitudes (complex bursts)) which occur within 3 ms (refractory period) and 15 ms (maximum ISI that defines the burst); and “neg” refers to the number of ISIs that have a negative effect on the CSI (violates either one or both of the rules for “pos”).

#### Sleep/Rest Trials Single Unit Properties

All single unit and LFP analyses were restricted to the first sleep recording of a given day (prior to running on familiar track). This was to examine baseline activity unrelated to experience and prevent cell duplication by using multiple epochs of the same session. The peak firing rate of a single unit during sleep/rest trial was defined as a maximal value of the firing rate curve (firing rate over time) calculated from the spike train by smoothing ISIs with a 5 SD Gaussian kernel. The mean firing rate of a single unit was calculated as total number of spikes emitted during the trial divided by that trial’s duration.

#### Ripple Event Detection

Ripple events were detected during rest periods bracketing the run sessions on the track when the mice sat quietly in a small familiar holding box. These periods are dominated by a quiet wake/slow wave sleep state, with occasional bouts of movement in both genotypes. On average we found no significant difference between the velocity of the mice across genotypes in the 3 s prior to ripple events (Controls: 0.081 ± 0.001 cm/s; p35 cKO: 0.094 ± 0.24 cm/s, *p* = 0.69). Ripple detection was performed using methods which have been previously described (Csicsvari et al., 1999). LFP traces were first band-pass filtered between 80 and 250 Hz using a 69 order Kaiser window zero-phase shift filter. The absolute value of a Hilbert transform (signal’s envelop) of the filtered LFP was smoothed with Gaussian window of 50 ms width and candidate ripple events were detected as periods where magnitude of the envelop exceeded 3 SD above its mean for a time longer than 30 ms. Initiation and termination of ripple events were identified by finding the periods in which the magnitude of envelop reached its mean value. Multi-unit activity (MUA) which was recorded from the identical TT as the one used as the LFP source was processed by instantaneous conversion to firing rate, and then smoothed. This method therefore enables the detection of firing bursts with same LFP threshold. If the detected candidate ripple events and MUA bursts did not coincide, they were excluded from the subsequent analysis.

#### Single Unit-Ripple Event Participation and Coactivity

In order to quantify single unit activity underlying ripple events, the event’s start and termination timestamps were used as boundaries for removing spikes occurred outside of the ripples. Then parameters such as number of spikes per ripple event per single unit, firing rate of any given single unit within SWR and percent of ripple events each single unit participated in (i.e., fired at least one spike) were calculated. Same calculation was performed while using timestamps of ripple event peaks and fixed interval (±200 ms) around the peak instead of start and stop timestamps yielding similar results. The “coactivity Z-score,” i.e., a likelihood of any given pair of pyramidal cells firing together during SWR events was calculated as described (Singer and Frank, 2009).

#### Burst Detection and Quantification

A burst was defined as at least two spikes fired within a 10 ms time bin. Burst detection and analysis were performed using MATLAB code previously described (Bakkum et al., 2014). Bursts were detected on a per-single unit basis yielding a set of properties, such as total number of bursts per trial, per minute, inter-burst interval, burst duration and number of spikes each burst consist of.

#### Power Spectral Density Calculation

Welch’s averaged modified periodogram method of spectral estimation (MATLAB’s pwelch function) was used to compute time-varying (0.63 s segments with 50% overlap) power spectra on 20 times (1600 Hz) decimated LFP traces. Any time periods when animal’s velocity dropped below 6 cm/s were excluded.

#### Pyramidal Cell Theta Wave Modulation

The relationship between spike firing times and theta band LFP phase was calculated as previously described (Siapas et al., 2005). Briefly, LFP traces were band-pass filtered through theta band (6–12 Hz, MATLAB eegfilt function), and instantaneous theta phase was derived from Hilbert—transformed signal. Setting the peaks of the theta oscillation as 0 degrees and the trough as 180 degrees, the spike phases were calculated using linear interpolation. This method is insensitive to theta wave asymmetry. The resulting phase values were converted to firing probability histograms, each incremented in 10-degree bin. Time periods during which an animal’s velocity was below 6 cm/s were excluded from this analysis. Significance of phase-related parameters including phase locking, preferred firing phase, strength of modulation, and mean resultant length (MRL) values and statistical comparison of phase values were computed using Circular Statistics Toolbox (Berens, 2009).

#### Statistics

Analyses were performed using custom written scripts in MATLAB (The MathWorks Inc., Natick, MA, USA), or in Prism 5 (GraphPad Software). Data were first tested for normality with the D’Agostino and Pearson omnibus normality test prior to further statistical analysis to determine whether parametric or non-parametric statistical tests were suitable. For pairwise comparisons, Student’s *t*-test or Mann Whitney tests were used as noted. All circular statistics were performed using the MATLAB Circular Statistics Toolbox. No statistical methods were used to pre-determine sample sizes, instead we opted to use group sizes of similar previously published studies (Tomar et al., 2015; Boehringer et al., 2017) to ensure variance was kept to a minimum between genotypes and cell quantities. Unless noted, all plots with error bars are reported as mean ± SEM and all samples are reported as number of mice (*N*) and number of cells (*n*).

## Results

**Figure 1:**
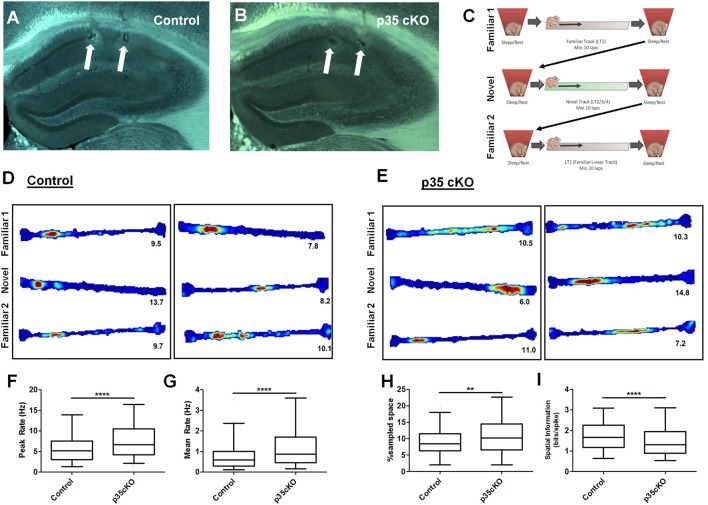
Increased excitability and poorer spatial coding in p35 cKO mice. Hippocampal anatomy was identical between **(A)** Control (*fl*p35) and **(B)** p35cKO (Cre^ER^/*fl*p35) mice. In both sections white arrows indicate electrolytic lesions marking tetrode (TT) locations in CA1. **(C)** Daily recording protocol used in these experiments. Mice were consecutively exposed to familiar and novel tracks daily, with each run session bracketed by rest/sleep periods. Example place cells recorded in **(D)** control and **(E)** p35cKO mice. Each box of three (top, middle, bottom) linear track (LT) rate maps show the place field for a single place cell in the order of familiar, novel and familiar recorded in the same session. Red color indicates peak firing rate, while blue color indicates a firing rate of zero. The number below each plot indicates the cell’s peak firing rate in that epoch. In p35 cKO mice, the **(F)** peak firing rate and the **(G)** mean firing rate during exploration of the familiar track is significantly increased. **(H)** Average CA1 place field size in the mutant mice is increased and **(I)** spatial information is decreased on the familiar track (All whisker plots indicate 5th percentile, median and 95th percentile; **p < 0.01; ****p < 0.0001).

To examine the impact of the loss of p35 in the adult brain we took advantage of mice engineered to allow the inducible post-natal knock-out of the gene encoding the protein (p35 cKO mice; Mishiba et al., 2014). p35 cKO mice, homozygous for the floxed p35 (*fl*p35) gene and carrying the tamoxifen-inducible Cre recombinase (Cre^ER^), and *fl*p35 control mice, lacking the Cre^ER^ transgene, were administered oral tamoxifen across 3 days to achieve the activation of Cre and deletion of the p35 gene in all cells in the Cre^ER^/*fl*p35 mice (Mishiba et al., 2014). These p35 cKO (*n* = 5) and floxed-p35 control mice (*n* = 4) were implanted with adjustable microdrives targeting TTs to the CA1 pyramidal cell layer. We recorded single unit activity and LFP during periods of rest and exploration of LTs (Figures 1A–C).

### Impaired Spatial Coding in CA1 Place Cells

Pyramidal cells in the hippocampus exhibit spatially receptive fields (“place fields”) that underlie a stable reliable map of an environment (O’Keefe and Dostrovsky, 1971). To examine the effects of the loss of p35 on cellular excitability and place coding, we recorded the activity of CA1 pyramidal cells as mutant (*N* = 5, *n* = 248) and control (*N* = 4, *n* = 272) mice ran laps on a familiar LT. While previous work had found no difference in exploration of a novel open field between these mutant and control groups (Mishiba et al., 2014), on our familiar track we observed a small but significant increase in the average velocity (Control: 4.2 ± 0.3 cm/s; p35 cKO: 5.7 ± 0.5 cm/s; *t*-test *p* = 0.04) in the mutants, perhaps reflecting their underlying differences in spatial learning (Mishiba et al., 2014).

We first characterized place cell activity. While place cells were clearly present in the mutant mice (Figures 1D,E), the spiking and spatial coding properties of these neurons were significantly altered. In p35 cKO mice we observed an increase in both the peak (Control (*N* = 4, *n* = 272): 5.89 ± 0.24 Hz; p35 cKO (*N* = 5, *n* = 248): 7.76 ± 0.29 Hz; Mann-Whitney, *p* < 0.0001; Figure 1F) and mean (Control (*N* = 4, *n* = 272): 0.80 ± 0.05 Hz; p35 cKO (*N* = 5, *n* = 248): 1.23 ± 0.07 Hz; Mann-Whitney, *p* < 0.0001; Figure 1G) firing rate during exploration. These increases in excitability resulted in larger place fields (Control (*N* = 4, *n* = 272): 15.7 ± 0.5% of sampled space; p35 cKO (*N* = 5, *n* = 248): 18.8 ± 0.7% of sampled space; Mann-Whitney, *p* = 0.0011; Figure 1H) and a significant decrease in the spatial information conveyed by the spiking of the neurons (Control (*N* = 4, *n* = 272): 1.75 ± 0.05 bits/spike; p35 cKO (*N* = 5, *n* = 248): 1.49 ± 0.05 bits/spike; Mann-Whitney, *p* < 0.0001; Figure 1I).

During the initial exploration of a novel context, CA1 pyramidal cells have elevated firing rates and the poorer spatial specificity (Nitz and McNaughton, 2004; Karlsson and Frank, 2008). To compare activity between control and p35 cKO mice during this encoding phase, we also recorded hippocampal activity during the first exploration of novel LTs. We observed a pattern of changes in the mutant mice that was identical to what we observed in the highly familiar track; higher mean firing rates (Control (*N* = 4, *n* = 161): 1.28 ± 0.13 Hz; p35 cKO (*N* = 5, *n* = 192): 1.77 ± 0.14 Hz; Mann-Whitney, *p* = 0.004) and poorer spatial coding (Control (*N* = 4, *n* = 161): 1.28 ± 0.07 bit/spike; p35 cKO (*N* = 5, *n* = 192): 1.01 ± 0.06 bit/spike; Mann-Whitney, *p* = 0.0013). These data suggest that the loss of p35 in the adult impairs the quality of the hippocampal spatial representation in both familiar and novel environments.

We next assessed the stability of the spatial coding in the mutant and control mice. Over time, there are changes in the spatial firing patterns of individual CA1 pyramidal cells even in a familiar environment (Mankin et al., 2012; Ziv et al., 2013). However, repeated exposures to the same context on the time scale of an hour or less typically results in a highly correlated spatial representation. To assess place field stability, we exposed mutant and control mice twice to the same familiar track within a single session and compared the spatial activity of neurons across these experiences. We found no significant difference in place field stability on the level of single neurons (Figures 1D,E; Pearson’s *r*; Con: 0.50 ± 0.03, cKO: 0.46 ± 0.02; Mann-Whitney *p* = 0.28) or in the average change in mean firing rate of the active neurons (Con: 0.30 ± 0.02, cKO: 0.34 ± 0.02; Mann-Whitney *p* = 0.10), suggesting the alterations in the excitability and coding did not impact the stability of spatial map on the timescale of hours.

### Excitability Increased During Sleep and Changes in Ripple Related Firing

**Figure 2:**
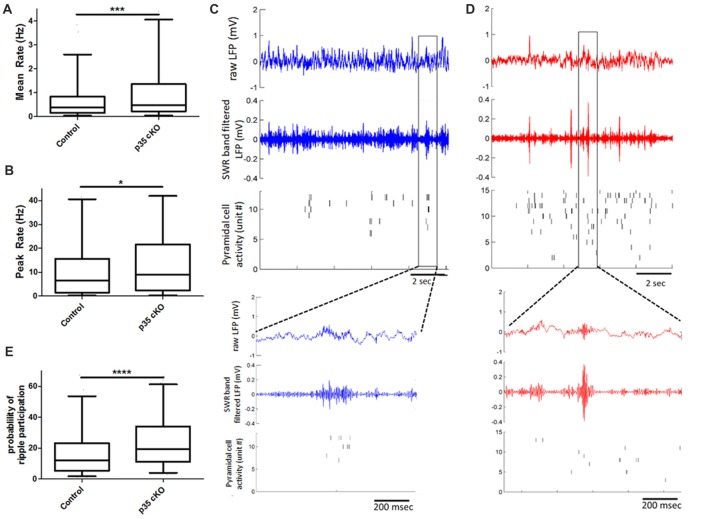
Increased pyramidal cell activity during sleep and ripples in p35 cKO mice. CA1 pyramidal cells in p35cKO mice have significantly higher **(A)** mean and** (B)** peak firing rates during baseline rest periods. **(C,D)** Local field potential (LFP) recordings shows prominent periodic sharp-wave ripples (SWRs) in both **(C)** control (blue) and **(D)** p35cKO (red) mice. Each plot shows 10 s of recording, top trace is the raw LFP, middle trace is LFP filtered in the SWR band (80–250 Hz) and bottom plot shows raster plots of spiking of individual simultaneously recorded CA1 pyramidal cell, each row is a single unit. Inset below is a zoomed in trace of 1 s of data showing example ripple event in each genotype. **(E)** CA1 pyramidal cells in p35cKO mice fired at least one spike in significantly greater fraction of ripple events (All whisker plots indicate 5th percentile, median and 95th percentile; *p < 0.05; ***p < 0.001; ****p < 0.0001).

To understand if the increased excitability we observed in the mutants during exploration carried over to periods of rest, we next examined single unit and LFP activity when mutant and control mice sat quietly in a small familiar enclosure. During these rest sessions, we observed a significant increase in both the peak (Control (*N* = 4, *n* = 351): 11.5 ± 0.7 Hz; p35 cKO (*N* = 5, *n* = 344): 13.5 ± 0.7 Hz; Mann-Whitney, *p* = 0.015) and mean firing rates (Control (*N* = 4, *n* = 351): 0.68 ± 0.04 Hz; p35 cKO (*N* = 5, *n* = 344): 1.03 ± 0.07 Hz; Mann-Whitney, *p* = 0.0005) of pyramidal cells in the p35cKO mice compared to controls (Figures 2A,B).

In the rest box, the hippocampal LFP is dominated by periodic SWR events that are thought to play a key role in organizing spiking for purposes of memory consolidation (Buzsáki, 2015). We found that the loss of p35 did not alter the occurrence of SWRs (Control (*N* = 4 mice, *n* = 10 sessions): 17.8 ± 1.4 ripples/min; p35 cKO (*N* = 5 mice, *n* = 14 sessions): 15.1 ± 1.2 ripples/min, *p* = 0.19), the peak oscillation frequency (Control (*N* = 4 mice, *n* = 10 sessions): 142.7 ± 3.6 Hz; p35 cKO (*N* = 5 mice, *n* = 14 sessions): 140.8 ± 2.3 Hz, *p* = 0.66) or amplitude of the events (Control (*N* = 4 mice, *n* = 10 sessions): 0.13 ± 0.02 mV; p35 cKO (*N* = 5 mice, *n* = 14 sessions): 0.15 ± 0.01 mV, *p* = 0.44; Figures 2C,D). However, when we examined pyramidal cell activity during SWRs, we found that neurons in the mutants participated in more events (Control (*N* = 4, *n* = 345 cells): 17.2 ± 0.9%; p35 cKO (*N* = 5, *n* = 344 cells): 24.8 ± 1.0%, Mann-Whitney test, *p* < 0.0001; Figure 2E) and on average fired more spikes per ripple event (Control (*N* = 4, *n* = 345 cells): 0.19 ± 0.02 spikes/ripples; p35 cKO (*N* = 5, *n* = 344 cells): 0.24 ± 0.01 spikes/ripples, Mann-Whitney test, *p* < 0.0001). This increase in ripple-related activity would lower the information content of SWR spikes in the mutants (Valero et al., 2017) and may be related to the memory deficits in these animals.

We next asked if this increase in ripple participation on the single cell level altered the organization of activity across the neuronal ensemble. We calculated co-activity of neurons during ripple events, restricting our analysis to neurons recorded on different TTs. We found that there was a significant increase in this pair-wise co-activity measure in the cKO mice (Control (*N* = 4, *n* = 5454 pairs) 0.14 ± 0.3, p35cKO (*N* = 5, *n* = 4062 pairs) 0.20 ± 0.03; Mann Whitney, *p* = 0.0009), suggesting the increase in excitability in the mutants impacts spiking organized across the population.

### Altered Bursting Properties

**Figure 3:**
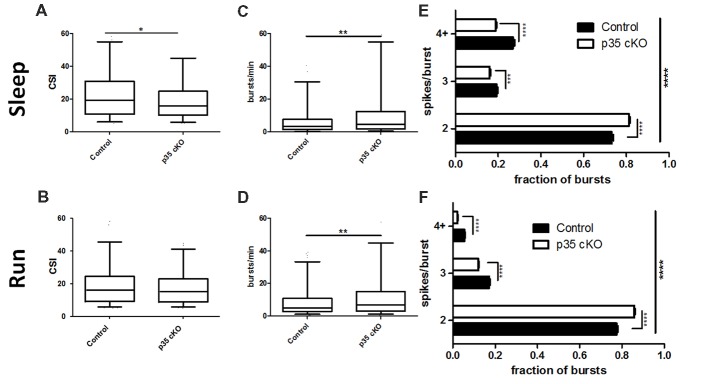
Reduction in complex spiking and burst length in p35 cKO mice. **(A)** During rest pyramidal cells in p35 cKO mice demonstrated a decrease in a measure of complex spiking, the complex spike index (CSI). **(B)** However, no significant difference in CSI could be noted during the run periods. CA1 pyramidal cells in p35 cKO mice generate more spike bursts during both **(C)** rest and **(D)** running. However, the average burst length was substantially shorter, as the proportion of two spike bursts was significantly increased in p35 cKO mice during** (E)** rest and **(F)** running (All whisker plots indicate 5th percentile, median and 95th percentile; *p < 0.05; **p < 0.01; ***p < 0.001; ****p < 0.0001).

Hippocampal pyramidal cells often fire in bursts, termed complex spikes, that are defined by consecutive spikes with short ISI (2–15 ms) and decreasing spike amplitude (Fox and Ranck, 1981). When we examined the CSI, a measure of the fraction of spikes belonging to these events (McHugh et al., 1996), we found a significant decrease in the mutants during rest periods (Control (*N* = 4, *n* = 351): 23.0 ± 0.8; p35 cKO (*N* = 5, *n* = 344): 19.2 ± 0.7; Mann-Whitney, *p* = 0.002), but not during exploration (Control (*N* = 4, *n* = 272): 19.3 ± 0.8; p35 cKO (*N* = 5, *n* = 248): 17.7 ± 0.7; Mann-Whitney, *p* = 0.18; Figures 3A,B). Given these changes we examined the bursting properties of CA1 pyramidal cells in greater detail.

In line with the observed increase in overall firing rate, but in contrast to the decrease in the CSI measure, pyramidal cells in the p35cKO mice exhibited an increase in the number of burst events per minute, both during rest and exploration (Rest: Control (*N* = 4, *n* = 351): 7.61 ± 0.68 bursts/min, p35 cKO (*N* = 5, *n* = 344): 11.89 ± 1.04 bursts/min, Mann Whitney, *p* = 0.007; Run: Control (*N* = 4, *n* = 302): 9.92 ± 0.90 bursts/min, p35 cKO (*N* = 5, *n* = 328): 13.07 ± 0.97 bursts/min, Mann Whitney, *p* = 0.0016; Figures 3C,D). To try to explain this seemingly paradoxical observation, we classified bursts by the number of spikes they contained and compared average burst length across genotypes, both during rest and running. We found that in both states, the average burst length was significantly shorter in the mutant mice, reflected as a significant increase in the fraction of 2 spike bursts and a significant decrease in bursts of 3 spikes or longer (Rest: 2-way ANOVA *F*_1,2_ (Genotype × Burst length) = 96.9, *p* < 0.0001; Bonferroni post-test Control × p35 cKO: 2 spike *p* < 0.0001, 3 spike *p* < 0.001, 4+ spike *p* < 0.0001; Run: 2-way ANOVA *F*_1,2_ (Genotype × Burst length) = 171.1, *p* < 0.0001; Bonferroni post-test Control × p35 cKO: 2 spike *p* < 0.0001, 3 spike *p* < 0.0001, 4+ spike *p* < 0.0001; Figures 3E,F).

### Change in Theta Modulation

**Figure 4:**
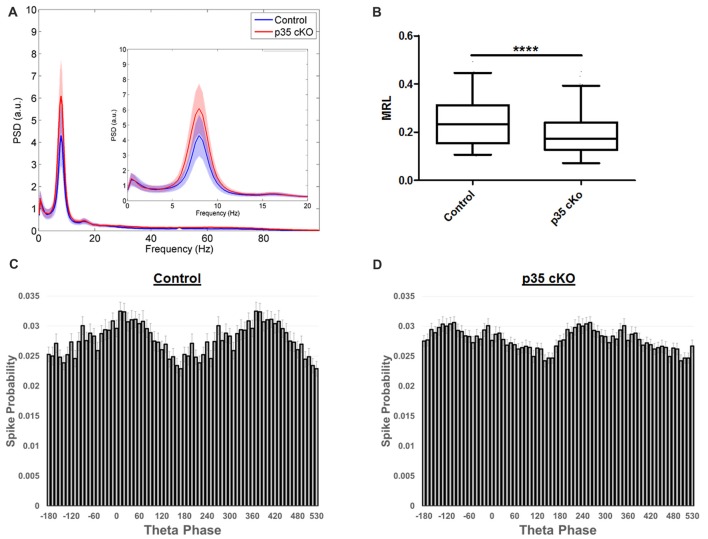
Altered theta modulation of CA1 pyramidal cells in p35 cKO mice. **(A)** Power spectrum density curves of CA1 LFP recorded during exploration of the familiar LT for control mice (blue, *N* = 4) and p35 cKO mice (*N* = 5). Inset shows an expanded view of the theta frequency range, no significant differences were found between the genotypes. **(B)** The depth of theta modulation (mean resultant length, MRL) of significantly theta modulated pyramidal cells is lower in the p35 cKO mice. A significant difference in the preferred theta phase of pyramidal cells spikes recorded in CA1 of **(C)** control and the **(D)** p35 cKO mice (Whisker plot indicates 5th percentile, median and 95th percentile; ****p < 0.0001).

During exploration, the hippocampal LFP is dominated by a prominent oscillation in the 6–12 Hz range, termed theta. This oscillation provides a network-wide temporal organization of pyramidal cell spiking, organizing the activity of the CA1 place cell ensemble (Buzsáki, 2002). Although we observed no significant changes in the power or frequency of theta recorded in the CA1 pyramidal cell layer LFP of p35 cKO mice (Figure 4A), there was a large reduction in the fraction of the pyramidal cells demonstrating significant modulation (Control (*N* = 4, *n* = 215): 75.8%, p35 cKO (*N* = 5, *n* = 286): 62.6%; *p* = 0.0008). Further, when we compared the modulation of the subset of significantly locked cells, we found both a decrease in the depth of modulation (MRL; Con (*N* = 4, *n* = 133): 0.241 ± 0.009, p35 cKO (*N* = 5, *n* = 169): 0.193 ± 0.007; Mann-Whitney *p* < 0.0001; Figure 4B) and a significant difference in preferred phase (circ_cm, *p* = 0.0077; Figures 4C,D). Thus, in addition to changes in the rate code present in CA1 place cells, the deletion of p35 also leads to profound deficits in the precise temporal coordination of spiking typically observed in the circuit.

## Discussion

Here, we provide the first analyses of the impact of dysregulation of the Cdk5/p35 complex on *in vivo* neurophysiology. p35 cKO mice showed significant alterations in both neuronal spiking and spatial coding. We observed an increase in pyramidal cell excitability which resulted in larger place fields with reduced spatial information, suggesting that the loss of p35 leads to impairments in the quality of hippocampal spatial representations in both familiar and novel contexts (Figures 1D–I). Further, while the loss of p35 did not alter the power or occurrence of the dominant hippocampal oscillations, theta and SWRs, it did lead to changes in how these rhythms organize spike output. Pyramidal cells in the mutant mice were less entrained by the theta oscillation (Figure 4B) and more promiscuous in their participation in ripples compared to the littermate controls (Figure 2E). Further, across the population, CA1 pyramidal cells in p35cKO mice had alterations in their bursting properties, generating more bursts, but of a significantly shorter burst length (Figure 3). Taken together, these data suggest that the loss of p35 is associated with excess and temporally disorganized pyramidal cell spiking, both during encoding and memory consolidation, perhaps underlying the severe spatial learning deficits observed in these mice (Mishiba et al., 2014).

The Cdk5/p35 complex has been shown to influence both pre- and post-synaptic neuronal function in the adult brain (Cheng and Ip, 2003; Cheung et al., 2006; Hawasli et al., 2007; Mita et al., 2016). These roles are reflected in the phenotypes reported in the p35 cKO mice, which include reduced CA1 pyramidal cell spine density and significantly lower Schaffer collateral evoked CA1 excitatory postsynaptic field potentials (Mishiba et al., 2014). Both these phenotypes could be expected to lower the overall excitability of CA1. It is important to note, however, that even with an inducible gene deletion it remains difficult to disambiguate whether these changes reflect the direct loss of p35 or rather, homeostatic changes resulting from alterations in cellular or network excitability. Nonetheless, we found significant increases in the average and peak firing rates of pyramidal cells in these mice, both during quiescence and locomotion, suggesting an overall increase in excitability when p35 is absent. This was particularly pronounced during SWR events, during which pyramidal cells in the mutant mice showed more spiking and increased event participation. This increase in excitability may be related to the impairment of LTD in these mice (Mishiba et al., 2014), as LTD selectively weakens synapses and has been implicated in memory and computational flexibility (Bear and Abraham, 1996).

One approach to deciphering the physiological phenotypes in the p35 cKO mice is to take advantage of the well-known biochemistry of the Cdk5/p35 complex to look for similar phenotypes in mice harboring mutations in related signaling molecules. Mice with forebrain-specific knock-out of the serine/threonine phosphatase calcineurin, known to mediate synaptic depression, also exhibit impaired glutamatergic LTD accompanied by an increase in SWR events with increased CA1 pyramidal cell participation (Zeng et al., 2001; Suh et al., 2013). Calcineurin is a phosphorylation target of the Cdk5/p35 complex and Cdk5 has been shown to function as a negative regulator of phosphatase activity (Hou et al., 2013), predicting an increase in calcineurin activity in the p35 cKO mice. This increase in calcineurin activity could also contribute to a LTD of GABA_A_ receptor mediated inhibition of CA1 pyramidal cells (Lu et al., 2000), further increasing cellular excitability. Together these results suggest that alterations in multiple forms of LTD may lead to inappropriate increases in the activity of CA1 pyramidal cells during SWRs.

A second point of biochemical and physiological convergence is observed between Cdk5/p35 and alpha Ca^2+^/calmodulin-dependent protein kinase II (αCaMKII). αCaMKII is involved in both synaptic plasticity and learning in the hippocampus (Elgersma et al., 2004). p35 is known to associate with αCamKII (Dhavan et al., 2002) and to downregulate kinase activity (Hosokawa et al., 2006). Further, in p35 cKO mice αCaMKII demonstrates enhanced phosphorylation at T286, an autophosphorylation site which increases the Ca^2+^ independent activity of the kinase (Mishiba et al., 2014). Place cells in mutant mice with a targeted point mutation of T286 that mimics this phosphorylation event (αCaMKII^T286D^ mice; Mayford et al., 1995) also demonstrated less precise spatial coding (Rotenberg et al., 1996). Interestingly, a second mutant strain, αCaMKII^T286A^ mice, with a mutation that abolishes this phosphorylation site exhibited decreased experience-dependent improvement in spatial coding, further implicating this pathway in place cell function (Cacucci et al., 2007). Finally, Cho et al. (2012) reported that place cells in αCaMKII^T305D^ mice, carrying a mutation that mimics inhibitory autophosphorylation of the kinase, exhibited altered bursting patterns manifested as shorter burst frequency. This was accompanied by a decrease in the number of spikes per burst compared to control mice, reminiscent of what we see in our data. Given that lowered levels of phosphorylation at T305 had been noted in p35 cKO mice (Mishiba et al., 2014), it may be speculated that p35 impacts hippocampal bursting via regulation of αCamKII activity and could contribute to the altered excitability of the hippocampal network. However, given the complexity of the possible phosphorylation sites which are considered in these studies, it is difficult to conclude a common mechanism between these observations.

Burst events reflect an integration of a neuron’s intrinsic excitability with the excitatory and inhibitory inputs it is receiving. Thus, it is important to consider changes in other cell types regulating CA1 pyramidal cell activity. Using cell-type specific optogenetics it has been shown that inhibition plays a key role in the structure and timing of pyramidal cell burst activities (Royer et al., 2012). Optogenetic silencing of dendrite-targeting SOM+ interneurons in CA1 greatly increase the probability of the occurrence of pyramidal cell burst firing, specifically favoring longer bursts. We find that in the absence of p35 there is an increase in the number of burst events per minute, but each burst is shorter in length (Figure 3), suggesting a possible enhancement of dendritic inhibition in this model. Further, a loss of Cdk5 function specifically in parvalbumin (PV) interneurons leads to an increase in GABAergic neurotransmission (Rudenko et al., 2015). Perisomatic targeting PV interneurons have a strong influence on the phase preferences of pyramidal cells during the theta oscillation (Royer et al., 2012). We observed a decrease in theta modulation across the pyramidal cell population in p35 cKO mice and a shift in preferred phase (Figure 4), suggesting some of the phenotypes we report here may result from activity changes in the inhibitory network. Thus, it is important to consider that p35 may have distinct cell-type specific roles in the diverse interneuron network found in CA1 (Klausberger and Somogyi, 2008) and future work targeting the gene deletion to subclasses of excitatory and inhibitory neurons in the circuit could have shed light on the origin of these phenotypes.

Here, we find that the deletion of the p35 gene in the adult mouse brain results in significant changes to the excitability and temporal coordination of hippocampal pyramidal cell activity. Given that the Cdk5/p35 complex has been implicated in a variety of disorders, including epilepsy (Wenzel et al., 2001; Putkonen et al., 2011) and AD (Lew et al., 1994; Patrick et al., 1999; Yoo and Lubec, 2001), these findings are helpful in deciphering the purpose of the continued expression of these proteins in postnatal neurons. Moreover, a phenotype similar to the decrease in theta modulation we observed in the p35 cKO mice has previously been reported in a transgenic mouse model of AD (Mably et al., 2017). This suggests that the loss of temporal organization may contribute to the memory deficits common to these lines. Further comparisons of *in vivo* phenotypes in converging disease models could help shed light on the links between the changes in biochemistry, synaptic physiology and behavior that define these disorders.

## Author Contributions

EK, TO and TM designed the study. EK and RB collected data. EK, RB, DP and TM analyzed data. EK, DP and TM prepared the figures. All authors discussed the results and wrote the manuscript.

## Conflict of Interest Statement

The authors declare that the research was conducted in the absence of any commercial or financial relationships that could be construed as a potential conflict of interest. The handling Editor declared a past supervisory role with one of the authors EK.
